# Evidence of Conformational Selection Driving the Formation of Ligand Binding Sites in Protein-Protein Interfaces

**DOI:** 10.1371/journal.pcbi.1003872

**Published:** 2014-10-02

**Authors:** Tanggis Bohnuud, Dima Kozakov, Sandor Vajda

**Affiliations:** 1 Program in Bioinformatics, Boston University, Boston, Massachusetts, United States of America; 2 Department of Biomedical Engineering, Boston University, Boston, Massachusetts, United States of America; 3 Department of Chemistry, Boston University, Boston, Massachusetts, United States of America; University of Houston, United States of America

## Abstract

Many protein-protein interactions (PPIs) are compelling targets for drug discovery, and in a number of cases can be disrupted by small molecules. The main goal of this study is to examine the mechanism of binding site formation in the interface region of proteins that are PPI targets by comparing ligand-free and ligand-bound structures. To avoid any potential bias, we focus on ensembles of ligand-free protein conformations obtained by nuclear magnetic resonance (NMR) techniques and deposited in the Protein Data Bank, rather than on ensembles specifically generated for this study. The measures used for structure comparison are based on detecting binding hot spots, i.e., protein regions that are major contributors to the binding free energy. The main tool of the analysis is computational solvent mapping, which explores the surface of proteins by docking a large number of small “probe” molecules. Although we consider conformational ensembles obtained by NMR techniques, the analysis is independent of the method used for generating the structures. Finding the energetically most important regions, mapping can identify binding site residues using ligand-free models based on NMR data. In addition, the method selects conformations that are similar to some peptide-bound or ligand-bound structure in terms of the properties of the binding site. This agrees with the conformational selection model of molecular recognition, which assumes such pre-existing conformations. The analysis also shows the maximum level of similarity between unbound and bound states that is achieved without any influence from a ligand. Further shift toward the bound structure assumes protein-peptide or protein-ligand interactions, either selecting higher energy conformations that are not part of the NMR ensemble, or leading to induced fit. Thus, forming the sites in protein-protein interfaces that bind peptides and can be targeted by small ligands always includes conformational selection, although other recognition mechanisms may also be involved.

## Author Summary

Many protein-protein interfaces (PPIs) are biologically compelling drug targets. Disrupting the interaction between two large proteins by a small inhibitor requires forming a high affinity binding site in the interface that generally can bind both peptides and drug-like compounds. Here we investigate whether such sites are induced by peptide or ligand binding, or already exist in the unbound state. The analysis requires comparing ligand-free and ligand-bound structures. To avoid any potential bias, we study ensembles of ligand-free protein conformations obtained by nuclear magnetic resonance (NMR) rather than generated by simulations. The analysis is based on computational solvent mapping, which explores the surface of the target protein by docking a large number of small “probe” molecules. Results show that ensembles of ligand-free models always include conformations that are fairly similar to some peptide-bound or ligand-bound structure in terms of the properties of the binding site. The analysis also identifies the models that are the most similar to a bound state, and shows the maximum level of similarity that is achieved without any influence from a ligand. While forming the binding site may require a combination of recognition mechanisms, there is preference for the spontaneous formation of bound-like structures.

## Introduction

Many protein-protein interactions (PPIs) are involved in disease pathways where therapeutic intervention could bring widespread benefit, and hence are biologically compelling targets for drug discovery [1], [2]. A number of systems are known for which small molecules inhibit the interaction between two proteins [3]–[6]. Some of the well studied targets include the complexes formed by MDM2 and p53 [7], Bcl-xL and the BAK protein [8], HPV-11 E2 and HPV-11 E1 [9], ZipA and FtsZ [10], HIV integrase and LEDGF/p75 [11], and IL-2 and its receptor IL-2Rα [12]. Apart from the IL-2/IL-2Rα system, in all complexes listed here one of the interacting proteins can be reduced to a peptide that binds on its own to the partner protein, and the small molecular inhibitors bind at the same site, mimicking some of the most important side chains of the peptide fragment. We note that most protein-protein interaction targets that can be disrupted by small drug-like molecules binding in the interface have this property. Since our focus is on the biophysical aspects of binding, in this paper we do not discriminate between peptide and non-peptide ligands, and thus ligand-bound protein will generally mean a complex co-crystallized either with a peptide or with a small molecule. Binding in the interface in both cases usually involves some conformational change. The main goal of this paper is to examine the mechanism of binding site formation in the interface region of proteins that are PPI targets. The conformational changes required for molecular recognition may occur due to two different mechanisms, known as induced-fit and conformational selection models [13]. The induced fit model treats the protein as if it exists in a single, stable conformation under given experimental conditions, and assumes that the structural plasticity in the molecule is induced by the binding [14]. In contrast, the conformational selection model describes a scenario in which the unbound protein exists in an ensemble of conformations some of which are similar to the ligand-bound state, and binding of the ligand shifts the distribution toward the bound state [13], [15], [16]. The two models are not mutually exclusive, and many recognition processes involve some elements of both mechanisms [14], [17]–[19].

To evidence conformational selection we need to show that some of the conformations in the free state resemble the ligand-bound structure [13]. Thus, the analysis requires an ensemble of ligand-free conformations and a measure that enables assessing the similarity between free and bound states. Ensembles of structures can be obtained by a variety of computational and experimental approaches. Molecular dynamics simulations show that transitional pockets may open up spontaneously at many different locations on the protein, some of them having the right size for ligand binding [20]–[22]. However, the required conformational transitions are rare on the time scales of ordinary simulations, and hence it is difficult to assess the significance of such sites. For example, it was recently shown that the known ligand binding sites in interfaces are more predisposed to surface pocket formation than the rest of the protein surface, but to obtain these results the simulations had to be biased toward pocket opening [23]. As alternatives to molecular dynamics, coarse-grained analysis tools such as elastic network models (ENMs) can be used to predict large-scale collective motions of proteins [24]–[26], but the method may be unable to capture highly localized changes.

While simulations tools are extremely useful for the characterization of molecular motion, they do not necessarily represent the best starting point for the analysis of recognition mechanisms, as the goal of increasing the number of conformational transitions for more significant results may unintentionally influence the conclusions. Thus, it is useful to consider conformational ensembles that are fully independent of the particular study. With this motivation in mind, in this work we consider already existing conformational ensembles, obtained by nuclear magnetic resonance (NMR) methods. The structures in such ensembles are low energy models that satisfy the highest number of NMR derived restraints. The ensembles are well documented as they have been deposited in the Protein Data Bank (PDB) [27]. Therefore, any analysis focusing on these structures should be unbiased and fully reproducible, thereby increasing the objectivity of conclusions. The structures within the ensembles show substantial variation, both in terms of the overall root mean square deviation (RMSD) and in the region of ligand binding. Previous works indicate that NMR derived structures can be very useful for the analysis of recognition mechanisms. For example, Lange and coworkers compared an ensemble of X-ray structures of ubiquitin, bound to different ubiquitin-binding proteins, with NMR structures of ubiquitin free in solution [28]. Results demonstrated that for each bound ubiquitin structure there is a member of the unbound ensemble that is structurally similar to it in the RMSD sense, thus giving strong support to the conformational selection model. However, as will be shown, our analysis will go further, as we develop a method that can find such structures without any information on the bound state or even on any potential ligand.

Once ensembles of free and ligand-bound conformations are available, we need an appropriate measure for comparing ligand-free and ligand-bound conformations in order to assess their similarity. The overall RMSD is clearly not suitable, since we are interested only in the changes around the ligand binding site. While one can calculate RMSD for the binding site residues, the latter are generally selected on the basis of their proximity to a bound ligand, which makes the results specific to a particular compound. The unique feature of this paper is that the measures used for structure comparison are based on binding hot spots, i.e., regions that are major contributors to the binding free energy. Binding hot spots are good binding sites in the general sense, i.e., without reference to any ligand [29]. The concept has been originally introduced in the context of mutating interface residues to alanine [30]. On the basis of this method, a residue is considered a hot spot if its mutation to alanine gives rise to a substantial drop in binding affinity. An alternative experimental method, more directly related to the binding of small ligands, is based on screening libraries of fragment-sized organic molecules for binding to the target protein. Since the binding of the small compounds is very weak, the interactions are most frequently detected by X-ray crystallography [31]–[33] or nuclear magnetic resonance (NMR) [34]. It was shown that the small “probe” ligands cluster at hot spots, and that the hit rate predicts the importance of the site [34]. While the existence of binding hot spots has been experimentally verified beyond doubt, there is no generally accepted explanation for their origin. Based on simulations, our hypothesis is that hot spots are distinguishable from other regions of the protein due to their concave topology combined with a mosaic-like pattern of hydrophobic and polar functionality [35]–[37]. Focusing on hot spots is particularly relevant for disrupting protein-protein complexes, since it requires finding a strong hot spot in the interface region of at least one of the component proteins [6]. We have shown that such interface hot spots can be reliably identified with the standard set of 16 small organic molecules that are used as probes in FTMap. Almost all probes have both hydrophobic and polar moieties, and many are relatively close side chain analogs [6], [38].

As the primary tools of our analysis, we rely on two algorithms called FTMap and FTSite. FTMap is a direct computational analog of the fragment screening experiments [39]. The method places each of 16 different small molecular probes on a dense grid around the protein and finds favorable positions using empirical energy functions. For each probe type, the individual probes are then clustered and the clusters are ranked on the basis of the average energy. Next, consensus clusters are identified as sites in which different probe clusters overlap. It has been extensively verified that FTMap reliably finds the binding hot spots identified by X-ray or NMR based screening [39]. The FTSite algorithm was developed from FTMap for the identification of binding sites [40] (see Methods). Since the binding site of proteins include a collection of hot spots, in FTSite we first select the hot spot that has the highest number of probe-protein contacts, and join it with the nearby hot spots. The amino acid residues in contact with the probes in this extended hot spot constitute the top ranked prediction of the ligand binding site. Extended hot spots with fewer probe-protein contacts define lower ranked binding site predictions. FTSite was shown to achieve substantially higher accuracy than any other current binding site prediction method, several of which were based on assessing the volume of binding pockets [40]. In fact, the number of probes bound to a site is a more direct and apparently more accurate measure of its expected binding affinity than the volume. The FTMap and FTSite algorithms were slightly modified for the purpose of this paper, primarily to increase the speed of the calculation and thus enable the method to analyze large ensembles of conformations (see Methods).

## Results

### Conformational ensembles

We have studied the five proteins listed in Table 1, with structures available in the Protein Data Bank (PDB) [27]. The table also includes some of the results that will be described later in the paper. The structures of these proteins have been determined by nuclear magnetic resonance (NMR) in the ligand-free state, resulting in conformational ensembles, and also in complexes with peptides or small molecular inhibitors (see Methods). The bound structures have been obtained either by X-ray crystallography or NMR. For each protein the ligand-free structures in the NMR ensemble show substantial conformational variation, both in terms of the overall RMSD and in the binding site. As will be further discussed, ligand binding substantially reduces the structural variation for each of the proteins (see Tables S1 through S4).

**Table 1:** Protein targets and summary of results.

Protein-Protein Complex Receptor/Ligand	Structures in PDB		Model with highest BSSC^c^	R^d^
	Unbound^a^	Bound (Ligand)^b^		
MDM2/p53	1z1m (24)	1ycr (p53 peptide)	Model 19 (2^nd^)	0.78
		1rv1 (Nutlin-2)	Model 19 (2^nd)^	0.72
		2lzg (piperidinone)	Model 9 (1^st^)	0.77
PSD-95 PDZ1/CRIPT	1iu2 (50)	1rgr (peptide)	Model 23 (1^st^)	0.92
MAGI-1 PDZ1/HPV16 E6	2kpk (20)	2kpl (peptide)	Model 9 (3^rd^)	0.77
EDC3/DCP2	4a53 (20)	4a54 (peptide)	Model 16 (2^nd^)	0.60
Bcl-xL/BAK, BAX, PUMA, BAD, etc.	2m03 (20)	2yxj (ABT-737)	Model 3 (1^st^)	0.85
		1bxl (BAK peptide)	Model 3 (1^st^)	0.72

a The number in parenthesis indicates the number of structures in the NMR ensemble.

b In parenthesis we indicate the ligand bound to the protein.

c BSSC denotes the bound-state similarity coefficient, which measures the similarity of each model to the bound state. The number of parenthesis is the rank based on the binding site hit rate (see Results).

d R denotes the correlation coefficient between the binding site hit rate and BSSC.

### Identification of binding sites

**Figure 1:**
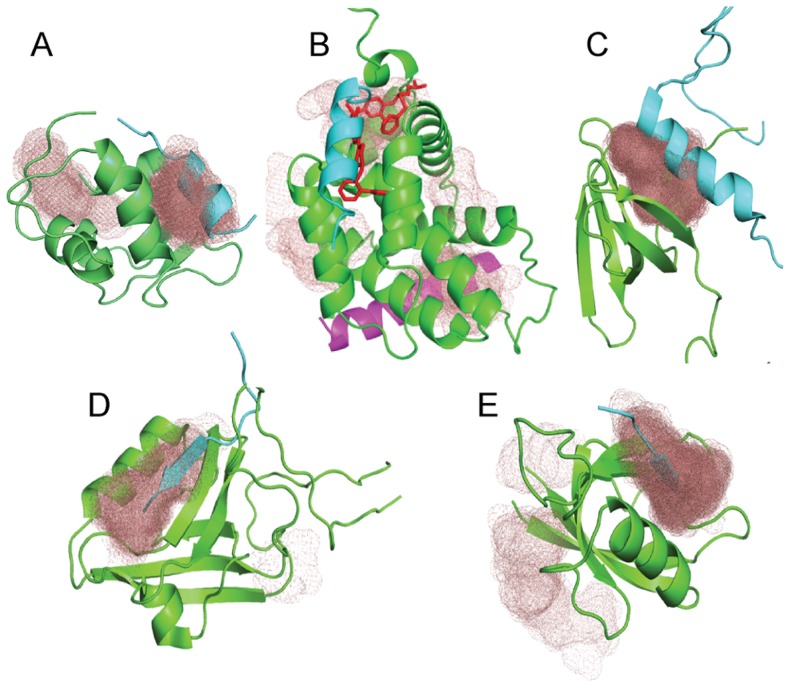
Identification of binding sites. **A.** Ligand-free MDM2 (1z1m, green) with p53 peptide (cyan) from the bound structure (3v3b). The top binding site predicted by FTSite (brown mesh) overlaps with the peptide in 18 of the 24 structures of 1z1m. **B.** Ligand-free Bcl-xL structure (2m03, green), with BAK peptide (cyan) from structure 1bxl, inhibitor ABT-737 (red sticks) from structure 2yxj, and the BIH SAHB peptide (magenta) binding to the close Bcl-xL homologue BAX (2k7w). The top predicted binding site (brown mesh) overlaps with the BAK peptide and ABT-737 in 6 of the 20 structures in 2m03, and with the BIH SAHB site in 9 of the 20 structures. **C.** Ligand-free EDC3 (4a53, green) with DCP2 peptide (cyan) from the structure 4a54. The top predicted binding site (brown mesh) overlaps with the peptide in all 20 structures in 4a53. **D.** Ligand-free MAGI1 PDZ1 (2kpk, green) with a C-terminal peptide of HPV16 E6 (cyan) from structure 2kpl. The top predicted binding site (brown mesh) overlaps with the peptide in 19 of the 20 structures in 2kpk. **E.** Ligand-free PSD95 PDZ1 (1iu2, green) with a peptide (cyan) from structure 1rgr. The top predicted binding site (brown mesh) overlaps with the peptide in 40 of the 50 structures in 1iu2).

Residues with any atom closer than 4 Å to any atom of the ligand in the bound structure were defined as binding site residues. Although for the five test proteins in Table 1 these residues are known, we explored whether the binding site can be found using only the ensemble of ligand-free structures, i.e., without any assumption on the ligand. We have previously developed the FTSite algorithm and server for the identification of binding sites on unbound protein structures [40]. The structures in the NMR derived ensembles of ligand-free proteins substantially differ from each other, which leads to variations in the binding sites predicted by FTSite. Nevertheless, selecting the site that is the top ranked prediction in the highest number of structures correctly identifies the ligand binding site for four of the five proteins in Table 1 (see Figure 1). The only exception is Bcl-xL. Mapping the NMR ensemble of ligand-free structures (PDB ID 2m03), the interaction site with the BAK peptide occurs as the top ranked binding site in only 6 of the 20 structures, and a different site is ranked first in the highest number of times (in 9 of the 20 structures). Although this second site is distinct from the canonical Bcl-xL binding groove, it was shown to be a highly functional peptide binding site in the BAX protein, a close homologue of Bcl-xL (see PDB ID 2k7w) [41]. In addition to determining the location of the main ligand binding sites, we can also use the FTSite results to find the binding site residues by selecting the residues that interact with probes in a substantial number of structures of the ensemble (see Methods).

### Binding site hit rate and bound state similarity coefficient

**Figure 2:**
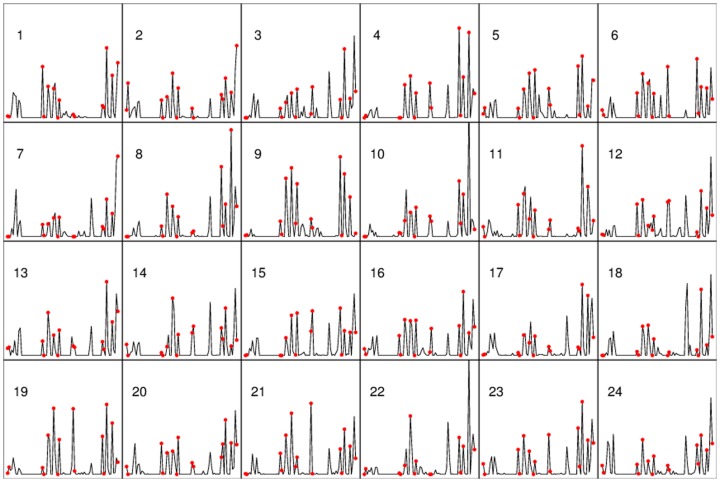
Mapping fingerprints of 24 unbound MDM2 structures. In each plot, horizontal axis, MDM2 residues (E25-Y104); vertical axis, percentage of probe-residue contacts (0–20%). Residues within 4 Å from the p53 peptide (PDB 1ycr) are marked with red dots.

As discussed, the residues in the binding site are likely to interact with probes in many structures. This is shown in Figure 2 for the 24 structures in the NMR ensemble of the ligand-free MDM2 (PDB ID 1z1m). For a more quantitative characterization of this relationship we introduce the concept of mapping fingerprint, defined as the number of probe-protein interactions for each residue, divided by the total number of interactions for all residues. For ligand-bound structures we also defined the ligand fingerprint, which is the number of ligand-protein interactions for each residue, divided by the total number of interactions for all residues. If the bound structure is an NMR ensemble, we calculated the average ligand fingerprint (see Methods). This is justified by the observation that the ligand fingerprints calculated for the different structures of the ensemble are highly correlated. As an example, Table S1 shows the pairwise correlation coefficients for models 1–5 of a ligand-bound MDM2. Table S1 also shows that the correlation is also high between the average ligand fingerprint and the ligand fingerprints of the individual models. Tables S2 through S4 show the latter type of correlation coefficients for the other proteins we have studied.

For a protein of *n* residues, for each unbound structure the mapping fingerprint define a vector of *n*-dimensional space, *X* = (*x_1_, x_2_,…,x_n_*), and the ligand fingerprint of the bound structure is also an *n*-dimensional vector, *Y* = (*y_1_, y_2_,…,y_n_*) (see an example in Figure S1). The correlation coefficient between these two vectors, given by
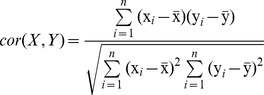
measures the similarity of the two structures in terms of the importance of binding site residues, and hence is defined as the bound-state similarity coefficient (BSSC). As will be shown, the structural variations in the ligand-free NMR ensembles cause substantial variations in BSSC, which can have values as high as 0.84 and as low as −0.04. One of the main questions considered here is how to identify, without information on any ligand, the particular ligand-free structure in the ensemble that is most similar to a ligand bound state in terms of BSSC. Our hypothesis is that the key predictor of this similarity is the binding site hit rate (HR), defined as the sum of probe-protein interactions for all binding site residues (apart from lysines and arginines in the binding site) divided by the total number of probe-protein interactions. The reason of not accounting for these two residues when calculating the binding site hit rate is that the positions of their side chains and hence their interactions with the probes are generally not very well defined, resulting in high level of uncertainty. The hypothesis assumes that well-formed pockets that are capable of binding specific ligands (e.g., peptides or small molecular inhibitors) also tend to bind a large number of probe molecules, and thus the level of non-specific binding is a predictor of specific binding ability. The mapping results support this hypothesis, and show that structures with the highest hit rates tend to be similar to some ligand-bound structure.

**Figure 3:**
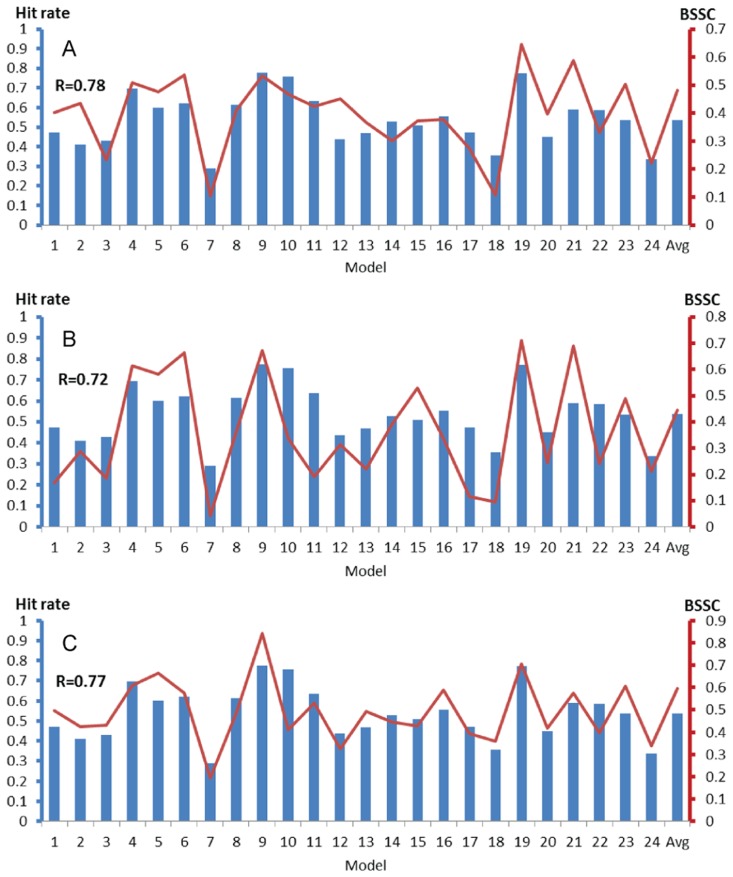
Binding site hit rates (blue columns, left axis) and BSSC (bound-state similarity coefficient) values (red lines, right axis) for the MDM2 ensemble (PDB ID 1z1m, 24 models). Horizontal axes list model numbers, with the last column showing the averaged binding site hit rate and BSSC value. BSSC values are defined for three different ligand-bound structures of MDM2. **A.** MDM2 bound to a p53 peptide (PDB ID 1ycr). **B.** MDM2 bound to the inhibitor Nutlin-2 (PDB ID 1rv1). **C.** MDM2 bound to a piperidinone derivative (PDB ID 2lzg).

Figure 3 shows the relationship between the binding site HR and BSSC for 24 NMR structures of ligand-free MDM2. The BSSC values were calculated for three different MDM2 structures bound to high affinity ligands, two of them small inhibitors and the third a peptide. The hit rates and BSSCs are also listed in Table S5. According to these results, model 9, which has the highest hit rate (0.78), also has the highest BSSC (0.84), the latter being based on the average ligand fingerprint of the NMR structure of MDM2 bound to a piperidinone inhibitor, PDB ID 2lzg [42]. The binding site in model 9 already shows some specificity, as it is substantially more similar to the piperidinone-bound structure than to the other two bound structures (with BSSC values of 0.53 and 0.67, respectively, see Table S5 and Figure 3). Model 19, which has the second highest hit rate (0.77), has the highest BSSC for two ligand-bound MDM2 structures, the first co-crystallized with a p53 peptide [43], and the second with the inhibitor Nutlin-2 [44]. The binding site in this model is less specific, as it is also similar to the piperidinone-bound structure, although less than model 9 is.

**Figure 4:**
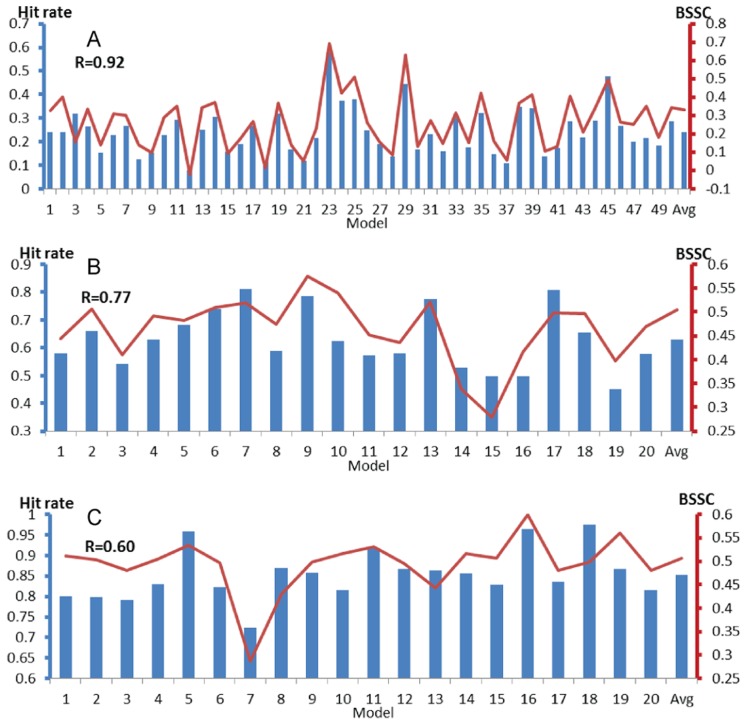
Binding site hit rates (blue columns, left axis) and BSSC (bound-state similarity coefficient) values (red lines, right axis) for the ligand-free NMR structures of PSD-95 PDZ1, MAGI-1 PDZ1, and EDC3. Horizontal axes list model numbers, with the last column showing the averaged binding site hit rate and BSSC value. **A.** Unbound PSD-95 PDZ1 ensemble (PDB ID 1iu2, 50 models). BSSC values are defined for a peptide-bound structure (PDB ID 1rgr). **B.** Unbound MAGI-1 PDZ1 ensemble (PDB ID 2kpk, 20 models). BSSC values are defined for a peptide-bound structure (PDB ID 2kpl). **C.** Unbound EDC3 ensemble (PDB ID 4a53, 20 models). BSSC values are defined for a peptide-bound structure (PDB ID 4a54).

**Figure 5:**
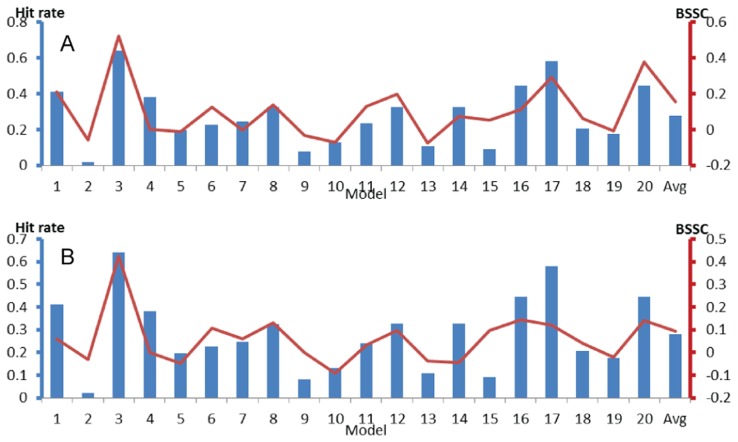
Binding site hit rates (blue columns, left axis) and BSSC (bound-state similarity coefficient) values (red lines, right axis) for the Bcl-xL ensemble (PDB ID 2m03, 20 models). Horizontal axes list model numbers, with the last column showing the averaged binding site hit rate and BSSC value. BSSC values are defined for three different ligand-bound structures of MDM2. **A.** MDM2 bound to the inhibitor ABT-737 (PDB ID 2yxj). **B.** MDM2 bound to a BAK peptide (PDB ID 1bxl).

As shown in Figure 4 and Tables S6 through S8, high hit rates also predict models that are similar to ligand-bound structures for PSD95 PDZ1, MAGI-1 PDZ1, and EDC3. For PSD-95 PDZ1, model 23 has the highest hit rate, and this model is also the most similar to the bound state defined by the X-ray structure of the PDZ1 domain co-crystallized with a cyclic peptide (Table S6). For MAGI-1 PDZ1, model 9 with the third highest hit rate (0.79) is the most similar to the peptide-bound structure 2kpl (Table S7). Models 7 and 17 have slightly higher hit rates, and are also fairly similar to the bound structure. Although at this point we have no known ligands that would yield ligand-bound structures with high level of similarity to these two models, their high hit rates imply that they have well-formed binding sites, and thus it is likely that ligands binding to these conformations will be found. For EDC3 the model most similar to the peptide bound state is model 16, which has the second highest hit rate (0.97), but the highest hit rate is not much different (0.98) (Table S8). Finally, according to the results for Bcl-xL, model 3 of the ensemble has the highest hit rate, and it is most similar to both structures bound to the inhibitor ABT-737 and a BAK peptide (Figure 5 and Table S9).

So far we have shown that some of the ligand-free models with high binding site hit rates tend to be similar to known bound structures. To further explore the relationship between hit rate and BSSC values, we note that, for an ensemble of *k* structures, both measures are given as *k*-vectors, and thus we can calculate their correlation coefficient. These correlation coefficients, listed in Table 1 and also shown in Figures 3, 4, and 5, were surprisingly large, between 0.60 and 0.92, indicating that the binding site hit rate can be used for the identification of structures that are likely to be the most similar to a ligand-bound state. We note that a similar method, called signal-to-noise ratio, has been recently introduced [45], and has been used for screening libraries of ligand cores against a library of receptor conformations without prior knowledge of specific pockets. Such methods are clearly very important if no ligand-bound structure of a protein is available, and hence one has to select the ligand-free structure that would be used for structure based ligand design.

## Discussion

We have originally introduced mapping fingerprints to show that the probes used for mapping interact with the same residues as the specific ligands of the protein, and that the relative importance of the residues is also conserved [36]. This property has been used for the identification of ligand binding sites, resulting in the FTSite method with demonstrated excellent performance [40]. Results show that comparing structures in terms of the similarity of their binding hot spots has two main advantages. First, although the mapping fingerprints for structures obtained by NMR show substantial variation among the members of the ensemble, indicating changes in the binding site, we were able to identify the binding site residues and to compare their importance in bound versus unbound structures without assuming any particular ligand. Second, the method identified the unbound structures in the ensemble that were likely to be the most similar ones to some ligand-bound structure, suggesting that the propensity of a site for binding small non-specific probes is highly correlated with the propensity of the same site for binding specific ligands. Although in this paper we considered only conformational ensembles obtained by nuclear magnetic resonance, the algorithm is independent of the method used for generating the structures, and can be applied to any ensemble of structures. Furthermore, here we focused on PPI targets in which the binding site and some small molecular inhibitors were already known, because comparing the unbound conformations identified by our method to ligand-bound structures demonstrated the power of the approach. However, we emphasize that the algorithm is based on the analysis of the protein structure, and does not require information on any potential ligand.

As shown in Figures 2 and S1, the residues interacting with ligands in the interface also interact with higher number of probe molecules. We have introduced the bound state similarity coefficient (BSSC), defined as the correlation coefficient between a mapping fingerprint of an unbound structure and the ligand fingerprint based on a ligand-bound structure, as the measure of similarity between binding sites in the two structures. We have observed that the BSSC values increase as the binding site acquires higher nonspecific binding affinity, measured in terms of the hit rate (HR), i.e., the number of probe atoms interacting with the residues in the site. The relationship between the hit rate and the bound state similarity coefficient was demonstrated by their consistently high correlation coefficients. As shown by some high BSSC values, the ensembles of NMR based models of ligand-free proteins always included some structures that were similar to ligand-bound states in terms of binding properties, indicating that the ligand binding sites within interface regions of PPI target proteins pre-exist in some of the unbound structures. This supports the conformational selection model, but the results also provide additional insights. Classical conformational selection assumes that proteins sample a vast conformational space and that higher energy states may bind the ligand [13], [15], [16], [46]. In the course of binding, because of favorable interactions with the ligand, these conformers get preferentially selected, and the population of protein microstates shifts in the direction of bound conformations [19]. Although our results do not contradict this paradigm, they suggest that the mechanism of changes leading to the formation of binding sites for small ligands in protein-protein interfaces goes beyond the conformational selection model. The 20 to 50 structures in the NMR ensembles deposited in the Protein Data Bank [27] are low energy models that satisfy the maximum number of NMR constraints, and clearly represent only a very small fraction of the conformational space. Thus, the conformational selection model does not fully explain why, in such small samples, we always find structures that have the pockets that are well formed for binding a large number of probes.

The spontaneous opening of pockets in protein-protein interfaces has been observed in simulations. Although Eyrisch and Helms found only short lived transient pockets, they noted that docking into these pockets generally led to conformations much closer to the complex structure than docking into the crystal structures of the free proteins [20]. Johnson and Karanicolas developed an improved computational methodology by adding a driving force towards conformations in which a surface pocket is present [23]. Starting from unbound protein structures, they have found conformational transitions that opened pockets at ligand binding sites in protein interfaces with little energetic cost to the protein. The ensembles of conformations generated with this biased approach structurally resembled known inhibitor-bound structures more closely than equivalent ensembles of unbiased conformations. Based on these results they concluded that the formation of such “druggable” sites is encoded in the protein surface [23]. Our analysis of the NMR ensembles fully supports this observation.

The potential origin of proteins having small ligand binding sites has been recently explored in a theoretical study by Gao and Skolnick [47], [48], who generated and analyzed two different libraries in artificial protein structures. The first library contained quasi-spherical, random protein structures packed in the same average spherical volume as proteins, but lacking backbone secondary structure and hydrogen bonding. While these structures had a statistically significant match to the global structures of native proteins, they were more densely packed and contained pockets that were too tiny to bind small molecules. The second library contained compact artificial structures with protein-like secondary structure. In contrast to the first library, these artificial proteins have pockets very similar to those of the native protein. This analysis shows that the biophysics of proteins, mainly their secondary structure, is likely to lead to the formation of broad specificity pockets. In fact, pockets are naturally formed when relatively rigid secondary structure elements are packed, e.g., at the ends of helical bundles, and even slight motion in these secondary structures may substantially change some of the pockets.

In spite of their very different methodologies, the studies by Johnson and Karanicolas [23] and by Gao and Skolnick [47], [48] arrive at the conclusion that the spontaneous formation of ligand binding sites leads to crude features with limited specificity that nevertheless restrict the range of complementary ligands, and additional smaller conformational changes then respond to details of a particular ligand. Our analysis of NMR structures shows the level of similarity between the binding sites in low energy unbound structures and those seen in the bound state. According to these results, BSSC can be as high as 0.84 for some complexes, but the typical value is closer to 0.6. Achieving similarity beyond this range assumes that there exist more similar but higher energy structures that are not present in the NMR ensemble, or that the similarity is further improved by induced fit. In any case, while forming binding sites in protein-protein interfaces may involve the combination of recognition mechanisms, conformational selection is an important part of the process.

Another important observation we made in this paper is that the ligand-free structures in the ensemble with the highest level of similarity to a ligand-bound structure also have high hit rates, i.e., they interact with a large number of small molecules used as probes for the mapping. Due to this property, both the structures and their putative ligand-binding sites can be identified by computational solvent mapping without reference to any particular ligand. Since these are the conformations that are most suitable for ligand design, the observation has clear practical significance. We have already used this property for selecting the most bound-like structures from ensembles computationally generated by different rotameric states of side chains in the binding site [6], [49]. However, the analysis of NMR structures of the ligand-free proteins, as described in this paper, provides a stronger and much more objective foundation for the method.

## Methods

### Selection of protein targets

We briefly describe the motivation for selecting the targets listed in Table 1, as well as the structural information used for each protein.

#### MDM2

The human version of the mouse double minute protein 2 (MDM2) is an important drug target for its role in binding and negatively regulating the tumor suppressor p53 [50]. We take the only available ligand-free structure of MDM2, which is an NMR derived ensemble of 24 models [50], and consider its residues 25–104. The distribution of NOEs and relaxation parameters confirmed that a significant portion of the domain is poorly structured. MDM2 structures have also been determined for complexes with a p53 peptide [43] and several small molecular inhibitors [42], [44]. These structures show that the two sub-domains of the protein must move apart in order to make place for ligands.

#### PDZ1 domain of PSD-95

The three PDZ domains of the postsynaptic density protein 95 (PSD-95) regulate signaling in glutaminergic neurons by modulating protein-protein interactions [51]. The solution structure of the PDZ1 domain has been determined using NMR [51], and the 50 lowest energy models have been deposited in the PDB. In most of these structures, the binding cleft is shallow and nondescript, consistent with the transient interactions aimed to bring proteins together to facilitate signaling, and then rapidly disperse. The structure of the PDZ1 domain has also been determined in a complex with a peptide designed for improved binding [52], resulting in better defined binding site and reduced structural variation.

#### PDZ1 domain of MAGI-1

The PDZ domains of membrane-associated guanylate kinase with inverted domains 1 (MAGI-1) interact with the E6 proteins of human papillomaviruses (HPVs) [53]. The solution structure of the MAGI-1 PDZ1 domain has been determined using NMR alone and bound to a peptide derived from the C-terminus of HPV16 E6 [53]. The comparison of these structures shows that the binding of the peptide induces quenching of high-frequency motions in the C-terminal tail of the PDZ domain.

#### LSm domain of yeast EDC3

The like-SM (LSm) domain of the enhancer of decapping 3 (EDC3) activator protein modulates the activity of the DCP1:DCP2 decapping complex, which catalyzes the removal of the mRNA 5′ cap [54]. The structure of the yeast EDC3 LSm domain has been determined using NMR both alone and in complex with a short helical leucine-rich motif of DCP2. Fromm et al. [54] deposited the 20 lowest energy structures for both the ligand-bound and ligand-free proteins.

**B-cell lymphoma-extra large (Bcl-xL).** In several tissues, DNA damage induces apoptosis via the stabilization of p53. Bcl-xL is an antiapoptotic protein, which sequester p53 [55], is overexpressed in many cancers, and thus has been pursued as a target for drug discovery. The structure of Bcl-xL has been determined ligand-free [55] and in complex with a variety of bound peptides and small molecule inhibitors [8], [56].

### Modifications of the FTMap and FTSite programs

The main tools of our analysis are the FTMap [39] and FTSite [40] programs. Both programs place small molecules as probes on the protein surface to determine consensus clusters that bind clusters of different probes. The ranking of consensus clusters is based on the number of probe clusters in FTMap, and on the number of non-bonded interactions between the protein and all probes in FTSite. Both FTMap and FTSite have been described previously [39], [40]. However, for this paper we introduced some changes in both algorithms, primarily to increase the computational speed. First, in the scoring function for the grid search, we use the simplified generalized Born (GB) type electrostatic term developed for the PIPER program [57], rather than a Poisson-Boltzmann model. Second, we do not perform off-grid local energy minimization, thereby reducing the computational efforts by almost an order of magnitude. All other details of the algorithms remain unchanged [39], [40].

### Selection of binding site residues

Using the FTSite results [40], for each residue we count the number of structures in which any atom of the residue is within 4 Å of the top prediction of the binding site, and rank the residues based on these counts. The selection of binding site residues starts with the top ranked residue, and we continue adding residues until 15% or more drop occurs in the count. We used a slightly different algorithm for Bcl-xL: the residues were ranked based on the number of structures in which they were found within 4 Å to any of the top three predicted binding sites, and added residues from this ranked list until a 50% drop in the count occurred. Although the cutoff rules introduced here may be specific to the proteins studied, it is general that the binding site residues are the ones that are close to the predicted binding sites in many structures of the NMR ensemble. The predicted and observed binding site residues are listed in Table S10.

### Calculation of the bound state similarity coefficient (BSSC)

BSSC for a ligand-free structure is the correlation coefficient between the mapping fingerprint, defined as the number of probe-protein interactions for each residue divided by the total number of interactions, and the ligand fingerprint of a bound structure, which is the number of ligand-protein interactions for each residue divided by the total number of interactions. If the bound structure is an NMR ensemble, BSSC is based on the average ligand fingerprint. As shown by the Tables S1 through S4, the ligand fingerprints are very similar within ensembles of such ligand-bound structures, and hence the average fingerprint is a valid measure of interactions.

## Supporting Information

Figure S1 Mapping fingerprints of MDM2 from unbound model 9 (blue), in comparison to ligand fingerprint calculated from a piperidinone bound structure (red). Horizontal axes list residues of MDM2 from Glu25 to TYR104 (unstructured regions removed before mapping analysis). Vertical axis shows the fraction of atom-atom interactions each protein residue makes with probe or ligand atoms.
(TIF)

Table S1 Validity of averaging fingerprints over bound structures solved by NMR. Pairwise correlation coefficients between the fingerprints for models 1–5 and the average fingerprint of the five ligand-bound MDM2 structures (PDB ID 2lzg).
(DOCX)

Table S2 Validity of averaging fingerprints over bound structures solved by NMR. Correlation coefficients between each fingerprint for models 1–22 and the average fingerprint of the 22 peptide-bound PSD-95 PDZ1 structures (PDB ID 1rgr).
(DOCX)

Table S3 Validity of averaging fingerprints over bound structures solved by NMR. Correlation coefficients between each fingerprint for models 1–20 and the average fingerprint from the ensemble of the 20 peptide-bound MAGI-1 PDZ1 structures (PDB ID 2kpl).
(DOCX)

Table S4 Validity of averaging fingerprints over bound structures solved by NMR. Correlation coefficients between the fingerprint for models 1–22 and the average fingerprint from the ensemble of the 22 peptide-bound EDC3 structures (PDB ID 4a54).
(DOCX)

Table S5 Binding site hit rates (HRs) and bound state similarity coefficients (BSSCs) for the ensemble of ligand-free MDM2 structures (PDB ID 1zlm). The BSSC values are calculated using the three ligand-bound structures with PDB IDs shown. The models are sorted based on the hit rate. The maximum value in each column is shown in bold.
(DOCX)

Table S6 Binding site hit rates and bound state similarity coefficients (BSSCs) for the ensemble of ligand-free PSD-95 PDZ1 structures (PDB ID 1iu2). The BSSC values are calculated using the ligand-bound structure with PDB IDs 1rgr. The models are sorted based on the hit rate. The maximum value in each column is shown in bold.
(DOCX)

Table S7 Binding site hit rates and bound state similarity coefficients (BSSCs) for the ensemble of ligand-free MAGI-1 PDZ1 structures (PDB ID 1kpk). The BSSC values are calculated using the ligand-bound structure with PDB IDs 2kpl. The models are sorted based on the hit rate. The maximum value in each column is shown in bold.
(DOCX)

Table S8 Binding site hit rates and bound state similarity coefficients (BSSCs) for the ensemble of ligand-free EDC3 structures (PDB ID 4a53). The BSSC values are calculated using the ligand-bound structure with PDB IDs 4a54. The models are sorted based on the hit rate. The maximum value in each column is shown in bold.
(DOCX)

Table S9 Binding site hit rates and bound state similarity coefficients (BSSCs) for the ensemble of ligand-free Bcl-xL structures (PDB ID 2m03). The BSSC values are calculated using the two ligand-bound structures with PDB IDs shown in the table. The models are sorted based on the hit rate. The maximum value in each column is shown in bold.
(DOCX)

Table S10 Predicted and observed binding site residues.
(DOCX)
