# Supplementary figures and images for: Evidence of Conformational Selection Driving the Formation of Ligand Binding Sites in Protein-Protein Interfaces
Source: PLoS Comput Biol. 2014 Oct 2;10(10):e1003872. doi: 10.1371/journal.pcbi.1003872 (PMC4183424; doi:10.1371/journal.pcbi.1003872)

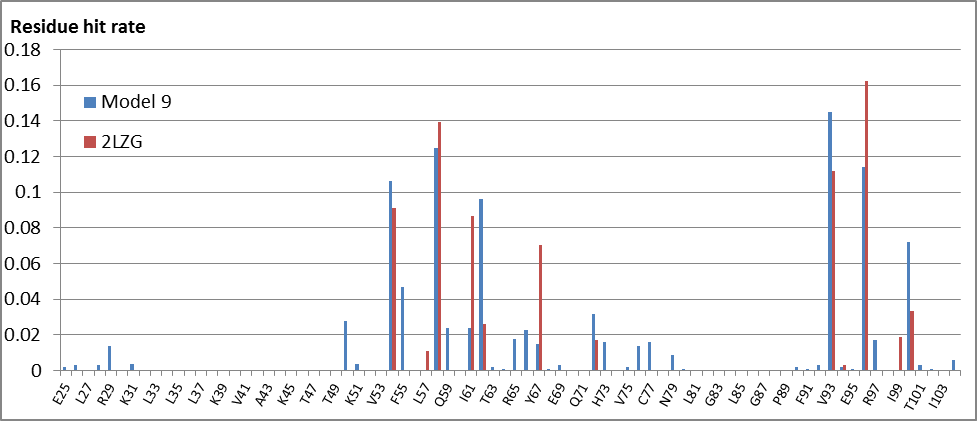

Supplement: Figure S1 — Mapping fingerprints of MDM2 from unbound model 9 (blue), in comparison to ligand fingerprint calculated from a piperidinone bound structure (red). Horizontal axes list residues of MDM2 from Glu25 to TYR104 (unstructured regions removed before mapping analysis). Vertical axis shows the fraction of atom-atom interactions each protein residue makes with probe or ligand atoms. (TIF) [file pcbi.1003872.s001.tif]
