# Supplementary material for: Evidence of Conformational Selection Driving the Formation of Ligand Binding Sites in Protein-Protein Interfaces
Source: PLoS Comput Biol. 2014 Oct 2;10(10):e1003872. doi: 10.1371/journal.pcbi.1003872 (PMC4183424; doi:10.1371/journal.pcbi.1003872)
Supplement: Table S1 — Validity of averaging fingerprints over bound structures solved by NMR. Pairwise correlation coefficients between the fingerprints for models 1–5 and the average fingerprint of the five ligand-bound MDM2 structures (PDB ID 2lzg). (DOCX) [file pcbi.1003872.s002.docx]

**Table S1. Binding site hit rates (HRs) and bound state similarity coefficients (BSSCs) for the ensemble of ligand-free MDM2 structures (PDB ID 1zlm). The BSSC values are calculated using the three ligand-bound structures with PDB IDs shown. The models are sorted based on the hit rate. The maximum value in each column is shown in bold.**

| **Model** | **HR** | **BSSC** | | |
| --- | --- | --- | --- | --- |
|  |  | **1ycr** | **1rv1** | **2lzg** |
| 9 | **0.78** | 0.53 | 0.67 | **0.84** |
| 19 | 0.77 | **0.65** | **0.71** | 0.71 |
| 10 | 0.76 | 0.47 | 0.34 | 0.41 |
| 4 | 0.70 | 0.51 | 0.61 | 0.61 |
| 11 | 0.64 | 0.42 | 0.19 | 0.53 |
| 6 | 0.62 | 0.54 | 0.66 | 0.58 |
| 8 | 0.61 | 0.41 | 0.36 | 0.49 |
| 5 | 0.60 | 0.48 | 0.58 | 0.66 |
| 21 | 0.59 | 0.59 | 0.69 | 0.57 |
| 22 | 0.59 | 0.33 | 0.24 | 0.40 |
| 16 | 0.55 | 0.38 | 0.34 | 0.59 |
| 23 | 0.54 | 0.50 | 0.49 | 0.60 |
| Average | 0.54 | 0.48 | 0.44 | 0.60 |
| 14 | 0.53 | 0.30 | 0.39 | 0.45 |
| 15 | 0.51 | 0.37 | 0.53 | 0.43 |
| 1 | 0.47 | 0.40 | 0.17 | 0.50 |
| 13 | 0.47 | 0.37 | 0.22 | 0.49 |
| 17 | 0.47 | 0.27 | 0.12 | 0.39 |
| 20 | 0.45 | 0.40 | 0.24 | 0.42 |
| 12 | 0.44 | 0.45 | 0.31 | 0.33 |
| 3 | 0.43 | 0.23 | 0.19 | 0.43 |
| 2 | 0.41 | 0.44 | 0.29 | 0.42 |
| 18 | 0.36 | 0.11 | 0.10 | 0.36 |
| 24 | 0.34 | 0.22 | 0.21 | 0.34 |
| 7 | 0.29 | 0.10 | 0.04 | 0.19 |
