# Supplementary material for: Evidence of Conformational Selection Driving the Formation of Ligand Binding Sites in Protein-Protein Interfaces
Source: PLoS Comput Biol. 2014 Oct 2;10(10):e1003872. doi: 10.1371/journal.pcbi.1003872 (PMC4183424; doi:10.1371/journal.pcbi.1003872)
Supplement: Table S2 — Validity of averaging fingerprints over bound structures solved by NMR. Correlation coefficients between each fingerprint for models 1–22 and the average fingerprint of the 22 peptide-bound PSD-95 PDZ1 structures (PDB ID 1rgr). (DOCX) [file pcbi.1003872.s003.docx]

**Table S2. Binding site hit rates and bound state similarity coefficients (BSSCs) for the ensemble of ligand-free PSD-95 PDZ1 structures (PDB ID 1iu2). The BSSC values are calculated using the ligand-bound structure with PDB IDs 1rgr. The models are sorted based on the hit rate. The maximum value in each column is shown in bold.**

| **Model** | **HR** | **BSSC** |
| --- | --- | --- |
| 23 | **0.58** | **0.69** |
| 45 | 0.48 | 0.50 |
| 29 | 0.45 | 0.63 |
| 24 | 0.38 | 0.42 |
| 25 | 0.38 | 0.51 |
| 38 | 0.35 | 0.37 |
| 39 | 0.34 | 0.41 |
| 3 | 0.32 | 0.16 |
| 19 | 0.32 | 0.37 |
| 35 | 0.32 | 0.42 |
| 14 | 0.31 | 0.37 |
| 33 | 0.3 | 0.32 |
| 11 | 0.29 | 0.35 |
| 42 | 0.29 | 0.41 |
| 44 | 0.29 | 0.34 |
| 50 | 0.29 | 0.34 |
| 4 | 0.27 | 0.33 |
| 7 | 0.27 | 0.3 |
| 17 | 0.27 | 0.27 |
| 46 | 0.27 | 0.26 |
| 13 | 0.25 | 0.34 |
| 26 | 0.25 | 0.26 |
| 1 | 0.24 | 0.33 |
| 2 | 0.24 | 0.4 |
| Average | 0.24 | 0.33 |
| 6 | 0.23 | 0.31 |
| 10 | 0.23 | 0.29 |
| 31 | 0.23 | 0.27 |
| 22 | 0.22 | 0.23 |
| 43 | 0.22 | 0.21 |
| 48 | 0.22 | 0.35 |
| 47 | 0.2 | 0.25 |
| 16 | 0.19 | 0.17 |
| 27 | 0.19 | 0.16 |
| 49 | 0.19 | 0.18 |
| 34 | 0.18 | 0.15 |
| 20 | 0.17 | 0.14 |
| 30 | 0.17 | 0.13 |
| 41 | 0.17 | 0.13 |
| 15 | 0.16 | 0.09 |
| 32 | 0.16 | 0.15 |
| 5 | 0.15 | 0.14 |
| 9 | 0.15 | 0.1 |
| 36 | 0.15 | 0.16 |
| 28 | 0.14 | 0.08 |
| 40 | 0.14 | 0.11 |
| 8 | 0.12 | 0.14 |
| 18 | 0.12 | 0.01 |
| 21 | 0.12 | 0.05 |
| 37 | 0.11 | 0.06 |
| 12 | 0.08 | -0.02 |
