# Supplementary material for: Evidence of Conformational Selection Driving the Formation of Ligand Binding Sites in Protein-Protein Interfaces
Source: PLoS Comput Biol. 2014 Oct 2;10(10):e1003872. doi: 10.1371/journal.pcbi.1003872 (PMC4183424; doi:10.1371/journal.pcbi.1003872)
Supplement: Table S3 — Validity of averaging fingerprints over bound structures solved by NMR. Correlation coefficients between each fingerprint for models 1–20 and the average fingerprint from the ensemble of the 20 peptide-bound MAGI-1 PDZ1 structures (PDB ID 2kpl). (DOCX) [file pcbi.1003872.s004.docx]

**Table S3.** **Binding site hit rates and bound state similarity coefficients (BSSCs) for the ensemble of ligand-free MAGI-1 PDZ1 structures (PDB ID 1kpk). The BSSC values are calculated using the ligand-bound structure with PDB IDs 2kpl. The models are sorted based on the hit rate. The maximum value in each column is shown in bold.**

| **Model** | **HR** | **BSSC** |
| --- | --- | --- |
| 7 | **0.81** | 0.52 |
| 17 | 0.81 | 0.50 |
| 9 | 0.79 | **0.58** |
| 13 | 0.78 | 0.52 |
| 6 | 0.74 | 0.51 |
| 5 | 0.68 | 0.48 |
| 2 | 0.66 | 0.51 |
| 18 | 0.66 | 0.50 |
| 4 | 0.63 | 0.49 |
| 10 | 0.63 | 0.54 |
| Average | 0.63 | 0.50 |
| 8 | 0.59 | 0.47 |
| 1 | 0.58 | 0.44 |
| 12 | 0.58 | 0.44 |
| 20 | 0.58 | 0.47 |
| 11 | 0.57 | 0.45 |
| 3 | 0.54 | 0.41 |
| 14 | 0.53 | 0.34 |
| 15 | 0.50 | 0.28 |
| 16 | 0.50 | 0.42 |
| 19 | 0.45 | 0.40 |
