# Supplementary material for: Evidence of Conformational Selection Driving the Formation of Ligand Binding Sites in Protein-Protein Interfaces
Source: PLoS Comput Biol. 2014 Oct 2;10(10):e1003872. doi: 10.1371/journal.pcbi.1003872 (PMC4183424; doi:10.1371/journal.pcbi.1003872)
Supplement: Table S4 — Validity of averaging fingerprints over bound structures solved by NMR. Correlation coefficients between the fingerprint for models 1–22 and the average fingerprint from the ensemble of the 22 peptide-bound EDC3 structures (PDB ID 4a54). (DOCX) [file pcbi.1003872.s005.docx]

**Table S4.** **Binding site hit rates and bound state similarity coefficients (BSSCs) for the ensemble of ligand-free EDC3 structures (PDB ID 4a53). The BSSC values are calculated using the ligand-bound structure with PDB IDs 4a54. The models are sorted based on the hit rate. The maximum value in each column is shown in bold.**

| **Model** | **HR** | **BSSC** |
| --- | --- | --- |
| 18 | **0.98** | 0.50 |
| 16 | 0.97 | **0.60** |
| 5 | 0.96 | 0.53 |
| 11 | 0.92 | 0.53 |
| 8 | 0.87 | 0.43 |
| 12 | 0.87 | 0.50 |
| 19 | 0.87 | 0.56 |
| 9 | 0.86 | 0.50 |
| 13 | 0.86 | 0.44 |
| 14 | 0.86 | 0.52 |
| Average | 0.85 | 0.51 |
| 17 | 0.84 | 0.48 |
| 4 | 0.83 | 0.51 |
| 15 | 0.83 | 0.51 |
| 6 | 0.82 | 0.50 |
| 10 | 0.82 | 0.52 |
| 20 | 0.82 | 0.48 |
| 1 | 0.80 | 0.51 |
| 2 | 0.80 | 0.50 |
| 3 | 0.79 | 0.48 |
| 7 | 0.72 | 0.29 |
