# Supplementary material for: Evidence of Conformational Selection Driving the Formation of Ligand Binding Sites in Protein-Protein Interfaces
Source: PLoS Comput Biol. 2014 Oct 2;10(10):e1003872. doi: 10.1371/journal.pcbi.1003872 (PMC4183424; doi:10.1371/journal.pcbi.1003872)
Supplement: Table S5 — Binding site hit rates (HRs) and bound state similarity coefficients (BSSCs) for the ensemble of ligand-free MDM2 structures (PDB ID 1zlm). The BSSC values are calculated using the three ligand-bound structures with PDB IDs shown. The models are sorted based on the hit rate. The maximum value in each column is shown in bold. (DOCX) [file pcbi.1003872.s006.docx]

**Table S5.** **Binding site hit rates and bound state similarity coefficients (BSSCs) for the ensemble of ligand-free Bcl-xL structures (PDB ID 2m03). The BSSC values are calculated using the two ligand-bound structures with PDB IDs shown in the table. The models are sorted based on the hit rate. The maximum value in each column is shown in bold.**

| **Model** | **HR** | **BSSC** | |
| --- | --- | --- | --- |
|  |  | **2yxj** | **1bxl** |
| 3 | **0.64** | **0.52** | **0.42** |
| 17 | 0.58 | 0.29 | 0.12 |
| 16 | 0.45 | 0.11 | 0.15 |
| 20 | 0.45 | 0.38 | 0.14 |
| 1 | 0.41 | 0.21 | 0.06 |
| 4 | 0.38 | 0.00 | 0.00 |
| 8 | 0.33 | 0.14 | 0.13 |
| 12 | 0.33 | 0.20 | 0.10 |
| 14 | 0.33 | 0.08 | -0.04 |
| Average | 0.28 | 0.16 | 0.09 |
| 7 | 0.25 | 0.00 | 0.06 |
| 11 | 0.24 | 0.13 | 0.03 |
| 6 | 0.23 | 0.13 | 0.11 |
| 18 | 0.21 | 0.06 | 0.04 |
| 5 | 0.20 | -0.01 | -0.05 |
| 19 | 0.18 | -0.01 | -0.02 |
| 10 | 0.13 | -0.07 | -0.09 |
| 13 | 0.11 | -0.07 | -0.04 |
| 15 | 0.09 | 0.05 | 0.10 |
| 9 | 0.08 | -0.03 | 0.00 |
| 2 | 0.02 | -0.06 | -0.03 |
