# Supplementary material for: Evidence of Conformational Selection Driving the Formation of Ligand Binding Sites in Protein-Protein Interfaces
Source: PLoS Comput Biol. 2014 Oct 2;10(10):e1003872. doi: 10.1371/journal.pcbi.1003872 (PMC4183424; doi:10.1371/journal.pcbi.1003872)
Supplement: Table S6 — Binding site hit rates and bound state similarity coefficients (BSSCs) for the ensemble of ligand-free PSD-95 PDZ1 structures (PDB ID 1iu2). The BSSC values are calculated using the ligand-bound structure with PDB IDs 1rgr. The models are sorted based on the hit rate. The maximum value in each column is shown in bold. (DOCX) [file pcbi.1003872.s007.docx]

**Table S6: Validity of averaging fingerprints over bound structures solved by NMR. Pairwise correlation coefficients between the fingerprints for models 1-5 and the average fingerprint of the 5 ligand-bound MDM2 structures (PDB ID 2lzg).**

| **Model** | **Model 1** | **Model 2** | **Model 3** | **Model 4** | **Model 5** | **Average** |
| --- | --- | --- | --- | --- | --- | --- |
| 1 |  | 0.794 | 0.91 | 0.84 | 0.963 | 0.954 |
| 2 |  |  | 0.874 | 0.897 | 0.804 | 0.922 |
| 3 |  |  |  | 0.857 | 0.876 | 0.953 |
| 4 |  |  |  |  | 0.86 | 0.943 |
| 5 |  |  |  |  |  | 0.954 |
