# Supplementary material for: Evidence of Conformational Selection Driving the Formation of Ligand Binding Sites in Protein-Protein Interfaces
Source: PLoS Comput Biol. 2014 Oct 2;10(10):e1003872. doi: 10.1371/journal.pcbi.1003872 (PMC4183424; doi:10.1371/journal.pcbi.1003872)
Supplement: Table S7 — Binding site hit rates and bound state similarity coefficients (BSSCs) for the ensemble of ligand-free MAGI-1 PDZ1 structures (PDB ID 1kpk). The BSSC values are calculated using the ligand-bound structure with PDB IDs 2kpl. The models are sorted based on the hit rate. The maximum value in each column is shown in bold. (DOCX) [file pcbi.1003872.s008.docx]

**Table S7: Validity of averaging fingerprints over bound structures solved by NMR. Correlation coefficients between each fingerprint for models 1-22 and the average fingerprint of the 22 peptide-bound PSD-95 PDZ1 structures (PDB ID 1rgr).**

| **Model** | **Correlation** |
| --- | --- |
| Model 1 | 0.991 |
| Model 2 | 0.989 |
| Model 3 | 0.983 |
| Model 4 | 0.988 |
| Model 5 | 0.984 |
| Model 6 | 0.982 |
| Model 7 | 0.971 |
| Model 8 | 0.988 |
| Model 9 | 0.989 |
| Model 10 | 0.993 |
| Model 11 | 0.966 |
| Model 12 | 0.976 |
| Model 13 | 0.992 |
| Model 14 | 0.989 |
| Model 15 | 0.973 |
| Model 16 | 0.978 |
| Model 17 | 0.971 |
| Model 18 | 0.98 |
| Model 19 | 0.982 |
| Model 20 | 0.975 |
| Model 21 | 0.982 |
| Model 22 | 0.992 |
