# Supplementary material for: Evidence of Conformational Selection Driving the Formation of Ligand Binding Sites in Protein-Protein Interfaces
Source: PLoS Comput Biol. 2014 Oct 2;10(10):e1003872. doi: 10.1371/journal.pcbi.1003872 (PMC4183424; doi:10.1371/journal.pcbi.1003872)
Supplement: Table S8 — Binding site hit rates and bound state similarity coefficients (BSSCs) for the ensemble of ligand-free EDC3 structures (PDB ID 4a53). The BSSC values are calculated using the ligand-bound structure with PDB IDs 4a54. The models are sorted based on the hit rate. The maximum value in each column is shown in bold. (DOCX) [file pcbi.1003872.s009.docx]

**Table S8: Validity of averaging fingerprints over bound structures solved by NMR. Correlation coefficients between each fingerprint for models 1-20 and the average fingerprint from the ensemble of the 20 peptide-bound MAGI-1 PDZ1 structures (PDB ID 2kpl).**

| **Model** | **Correlation** |
| --- | --- |
| Model 1 | 0.915 |
| Model 2 | 0.757 |
| Model 3 | 0.881 |
| Model 4 | 0.861 |
| Model 5 | 0.888 |
| Model 6 | 0.899 |
| Model 7 | 0.891 |
| Model 8 | 0.872 |
| Model 9 | 0.931 |
| Model 10 | 0.907 |
| Model 11 | 0.909 |
| Model 12 | 0.885 |
| Model 13 | 0.895 |
| Model 14 | 0.932 |
| Model 15 | 0.928 |
| Model 16 | 0.856 |
| Model 17 | 0.934 |
| Model 18 | 0.921 |
| Model 19 | 0.873 |
| Model 20 | 0.902 |
