# Supplementary material for: Evidence of Conformational Selection Driving the Formation of Ligand Binding Sites in Protein-Protein Interfaces
Source: PLoS Comput Biol. 2014 Oct 2;10(10):e1003872. doi: 10.1371/journal.pcbi.1003872 (PMC4183424; doi:10.1371/journal.pcbi.1003872)
Supplement: Table S9 — Binding site hit rates and bound state similarity coefficients (BSSCs) for the ensemble of ligand-free Bcl-xL structures (PDB ID 2m03). The BSSC values are calculated using the two ligand-bound structures with PDB IDs shown in the table. The models are sorted based on the hit rate. The maximum value in each column is shown in bold. (DOCX) [file pcbi.1003872.s010.docx]

**Table S9: Validity of averaging fingerprints over bound structures solved by NMR. Correlation coefficients between the fingerprint for models 1-22 and the average fingerprint from the ensemble of the 22 peptide-bound EDC3 structures (PDB ID 4a54).**

| **Model** | **Correlation** |
| --- | --- |
| Model 1 | 0.981 |
| Model 2 | 0.984 |
| Model 3 | 0.893 |
| Model 4 | 0.897 |
| Model 5 | 0.908 |
| Model 6 | 0.971 |
| Model 7 | 0.86 |
| Model 8 | 0.979 |
| Model 9 | 0.951 |
| Model 10 | 0.994 |
| Model 11 | 0.9 |
| Model 12 | 0.925 |
| Model 13 | 0.949 |
| Model 14 | 0.967 |
| Model 15 | 0.971 |
| Model 16 | 0.948 |
| Model 17 | 0.954 |
| Model 18 | 0.944 |
| Model 19 | 0.965 |
| Model 20 | 0.986 |
| Model 21 | 0.843 |
| Model 22 | 0.962 |
