# Supplementary material for: Evidence of Conformational Selection Driving the Formation of Ligand Binding Sites in Protein-Protein Interfaces
Source: PLoS Comput Biol. 2014 Oct 2;10(10):e1003872. doi: 10.1371/journal.pcbi.1003872 (PMC4183424; doi:10.1371/journal.pcbi.1003872)
Supplement: Table S10 — Predicted and observed binding site residues. (DOCX) [file pcbi.1003872.s011.docx]

**Table S10. Predicted and observed binding site residues.**

| **System (ligand)** | **Binding site residues predicted from unbound ensemble** | **Binding site residues observed in ligand-bound structures^a^** | **Precision^b^** |
| --- | --- | --- | --- |
| MDM2 (Nutlin-2) | L54 F55 G58 Q59 M62 Q72 H73 V93 K94 H96 Y100 | L54 F55 L57 G58 I61 M62 Y67 Q72 H73 V75 F86 F91 V93 H96 I99 Y100 | 81.82 |
| MDM2 (peptide) |  | E25 T26 M50 K51 L54 F55 L57 G58 I61 M62 Y67 Q71 Q72 H73 V75 F86 F91 V93 K94 H96 I99 Y100 I103 Y104 | 90.91 |
| PSD-95 PDZ1^c^ | S13 G14 L15 G16 F17 S18 I19 I40 V74 L77 K78 | R70 S73 G74 L75 G76 F77 S78 I79 A80 G81 N85 P86 H87 I88 F95 I96 T97 K98 I100 H130 S131 V134 L137 K138 V144 | 100.00 |
| MAGI-1 PDZ1 | K22 R25 F27 F29 T30 V31 H77 V81 F84 Q85 | G26 F27 G28 F29 T30 V31 V32 G33 D35 E36 D38 E39 F40 L41 Q42 K44 V47 H77 V80 V81 F84 Q85 P107 D108 T112 S113 L114 V115 T116 | 80.00 |
| EDC3 | V3 F6 V11 I25 L35 T49 F28 K50 I52 L55 R56 I57 | M-1 M1 S2 V3 F6 F28 S33 T49 I52 K53 D54 L55 R56 I57 K70 | 66.67 |
| Bcl-xL^c^ (ABT-737) | A53 E56 A57 E60 F61 R64 Y65 R66 A68 F69 L72 E93 L94 R96 D97 G98 N100 W101 G102 I104 V105 A106 I146 Q147 G150 G151 T154 F155 L158 Y159 G160 N161 N162 A165 R168 R173 L174 | A93 E96 F97 R100 Y101 A104 F105 L108 V126 E129 L130 N136 W137 G138 R139 V141 A142 S145 F146 A149 F191 L194 Y195 N197 | 78.13^d^ |
| Bcl-xL^c^ (peptide) |  | E96 F97 R100 Y101 A104 F105 L108 Q111 L112 Q125 V126 E129 L130 R132 R139 A142 F146 L194 Y195 A199 A200 S203 R204 | 40.00^d^ |
| ^a^Binding site residues are defined as residues having any atom within 4 Å of any ligand atom  ^b^Precision is calculated as percentage of true positives in total positive prediction  ^c^The residue numbering between unbound and bound structures is off by 60 (PSD-95 PDZ1) and by 36 (Pcl-xL); the numbers here adhere to the PDB structures  ^d^Some terminal residues are predicted to be in binding site, but are missing in the ligand-bound structures of Bcl-xL; these residues are excluded from the calculation of precision | | | |
